# P358: Application of quality standards obstetric neonatal emergencies in benin

**DOI:** 10.1186/2047-2994-2-S1-P358

**Published:** 2013-06-20

**Authors:** TA Ahoyo, GA Attolou, S Assavedo, C Lodjou, GM Amoussou, KAD Gazard

**Affiliations:** 1 Health Ministry of Benin, Cotonou, Benin

## Introduction

The development and proper implementation of norms and standards in health care helps to improve quality in hospitals. In this perspective, a situational analysis pilot was decided by the Minister of Health of Benin at the request of WAHO / RIPAQS to harmonize standards and quality assurance standards of care in African hospitals.

## Objectives

To evaluate the status of norms and standards and their applicabilities especially in obstetric and neonatal emergencies in Benin.

## Methods

A pilot cross-sectinal multicenter study took into account a purposeful sample of 17 health facilities in Benin. Data were collected by interview live via focus groups and direct observation using six (6) questionnaires developed by the RIPAQS supported by WAHO and offered to each participating country. The data were processed by SPSS software EpiData 3.1 and 19.1. A rating scale from 0 to 100 was used to assess the level of quality.

## Results

An average score around 50% of performance is observed. Some highlights: 79% for the application of standards and delivery practices in obstetric and neonatal emergencies, 54% existence of hzgiene measures for infant and maternal health, 75% of maternal care and the newborn in the postpartum period, some weaknesses: 65% of staff over 50 years, 75% of staff are overloaded, 40% lack of communication at all levels.

## Conclusion

The dysfunctions in the development and application of standards and norms were recorded in particular the lack of appropriate institutional and regulatory frameworks. The hospital staff is aging and renewed with a little extra work. Financial and technical coaching and a harmonization of norms and standards are recommended.

## Disclosure of interest

None declared

